# ADHD symptoms are associated with bully victimization in non-clinical populations too

**DOI:** 10.1192/j.eurpsy.2024.940

**Published:** 2024-08-27

**Authors:** M. R. Glans, S. Bejerot

**Affiliations:** ^1^ Örebro University, School of Medical Science; ^2^Örebro University, School of Medical Sciences, Örebro, Sweden

## Abstract

**Introduction:**

Individuals with ADHD are at higher risk of being bullied than individuals without ADHD^1,2,3^ Over the past decades, there has been a shift from a categorical to a dimensional conceptualization of ADHD^4.^ It remains unknown if the association between ADHD and bullying also extends to non-clinical populations.

**Objectives:**

To assess if subclinical ADHD symptoms associates with bully victimization in childhood and adolescence.

**Methods:**

1557 non-clinical adults completed the 6-item Adult Self-Report Scale Screener (ASRS) and answered questions concerning bully victimization. ADHD and ASD diagnoses served as exclusion criteria. Prevalence rates of bully victimization (defined as bullied ≥twice monthly) were compared at different time periods between those with- and without a positive ASRS-screener (cut-off score ≥4/6) by chi-square tests. Moreover, logistic regression evaluated the association while adjusting for candidate covariates age and sex.

**Results:**

Out of the total sample 1332 individuals (mean age=42, 60% female) scored negative and 217 individuals (mean age=36, 70% female) scored positive on the ASRS-screener while 8 had missing data on age or sex. Prevalence rates of bully victimization comparing those with- and without a positive score were as following; 20% vs 11%, p<.001 at 7-9 years, 26% vs 15%, p<.001 at 10-12 years, 20% vs 13%, p=.005 at 13-15 years and 6% vs 2%, p=.002 at 16-18 years. The statistically significant associations seen in the prevalence comparisons up until working life remained in the logistic regression models.

**Conclusions:**

More pronounced subclinical ADHD symptoms were associated with approximately twice as high prevalence of bully victimization in childhood and adolescence. Thus, ADHD characteristics appear to have serious consequences across the full clinical and non-clinical parts of the spectrum.

**Disclosure of Interest:**

None Declared

